# Additional data on damage reduction strategies against chemical accidents by using a mitigation barrier in Korean chemical risk management

**DOI:** 10.1016/j.dib.2018.08.138

**Published:** 2018-08-31

**Authors:** Byeonggil Lyu, Kwanghee Lee, Taejong Kim, Hyungtae Cho, Seungsik Cho, Il Moon

**Affiliations:** Department of Chemical and Biomolecular Engineering, Yonsei University, 50 Yonsei-ro, Seodaemun-gu, Seoul 03722, Republic of Korea

## Abstract

This paper describes data of post-release mitigation strategy and its effect for chemical process. The data in this paper is associated with the article entitled “Damage reduction strategies against chemical accidents by using a mitigation barrier in Korean chemical risk management”. The data includes computational fluid dynamics (CFD) simulation result for vapor cloud explosion accident scenarios. Simulations with suggested mitigation strategy and without the mitigation strategy were conducted. The data is expected to good reference for developing chemical plant mitigation plan.

## Specifications table

**Table:** 

Subject area	*Chemical Engineering*
More specific subject area	*Chemical Process Systems Engineering Safety*
Type of data	*Figures and Tables*
How data was acquired	*Computational fluid dynamics (CFD) simulation by software, FLACS v10.3*
Data format	*Raw*
Experimental factors	*Operating condition, Leak hole size, Leak time*
Experimental features	*Case study, Performance evaluation*
Data source location	*Department of Chemical and Biomolecular Engineering, Yonsei University*
Data accessibility	*Data with this article*

## Value of the data

• Simulated the effect of mitigation barrier which constructed in real plant.
• Assumed vapor cloud explosion accident that may occur under actual process conditions.
• Provided data helps to develop post-release mitigation plan in real plant.

## 1 Data description

In this data article, we share simulation result of vapor cloud explosion (VCE) accident. VCE accident is also important consideration for developing chemical accident mitigation strategy. The article entitled “Damage reduction strategies against chemical accidents by using a mitigation barrier in Korean chemical risk management” [1] includes only diffusion of materials, not including the effect of VCE. In order to provide more accident simulation information, this paper includes VCE simulation data. Simulations were conducted for each accident scenarios when there is no barrier and when there is a barrier. In this data, accident scenario information and CFD simulation result are illustrated.

## 2 Simulation design, methods and result

### 2.1 Estimation of explosion accident scenario

Simulation conditions for column are shown in Table 1. Operating conditions and leak information is described. The conditions are estimated following Korean government standards, and the wind condition assumed annual average speed and worst direction. Lower flammable limit (LFL) and Upper flammable limit (UFL) of xylene is also shown in Table 2.

**Table 1:** Column input data.

Parameter	Value
Material	Xylene
Operating temperature	217.5 °C
Operating pressure	4.53 kg/cm^2^ g
Leak point height	29 m
Leak hole diameter	0.1 m
Distance to public	206.33 m
Leak flow rate	13.9 kg/s
Wind	3.1 m/s to public

**Table 2:** LFL and UFL information of Xylene isomers.

**Material**	**LFL (%)**	**UFL (%)**
**m-Xylene**	1.1	7.0
**o-Xylene**	0.9	6.7
**p-Xylene**	1.0	6.0

**Fig. 1:**
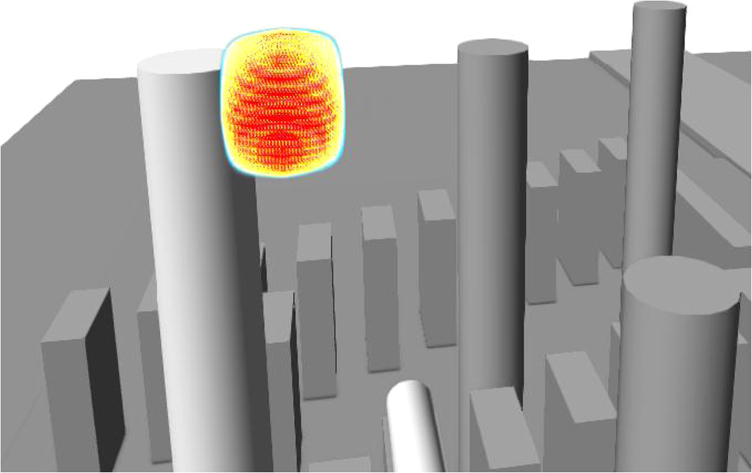
The xylene concentration area between LFL and UFL.

**Fig. 2:**
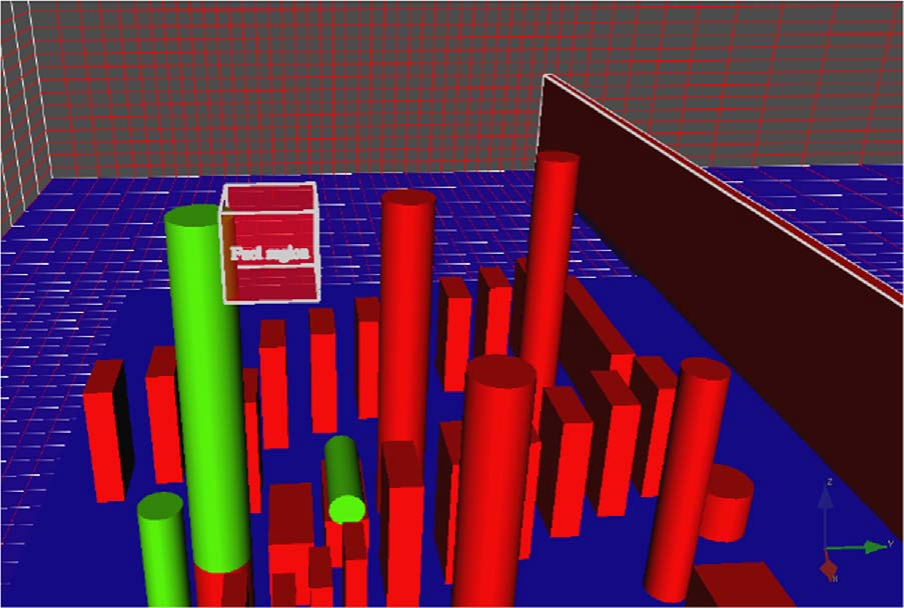
Explosion geometry.

The explosion accident occurs when composition of xylene is within LFL and UFL. Fig. 1 shows the area with the concentration between LFL and UFL, showing 3–6%. Fig. 2 shows the explosion range geometry which is constructed based on Fig. 1.

### 2.2 Vapor cloud explosion simulation

**Fig. 3:**
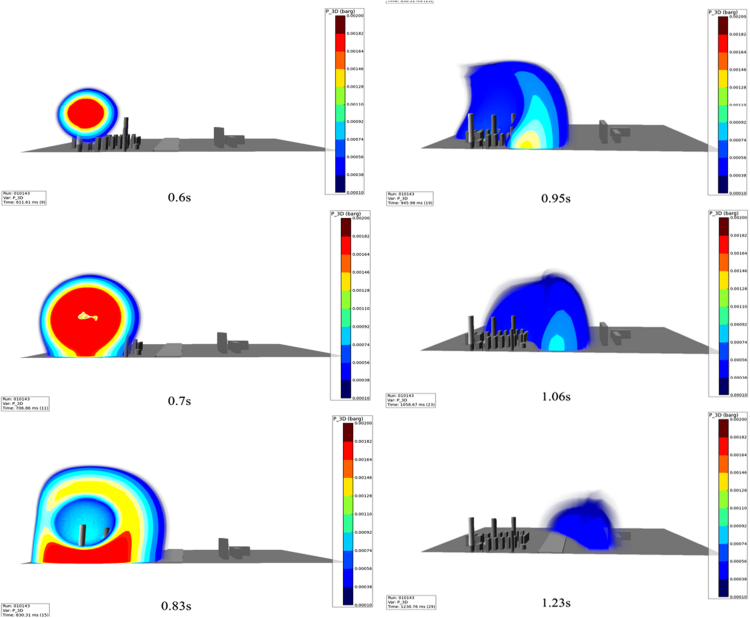
VCE without mitigation barrier.

**Fig. 4:**
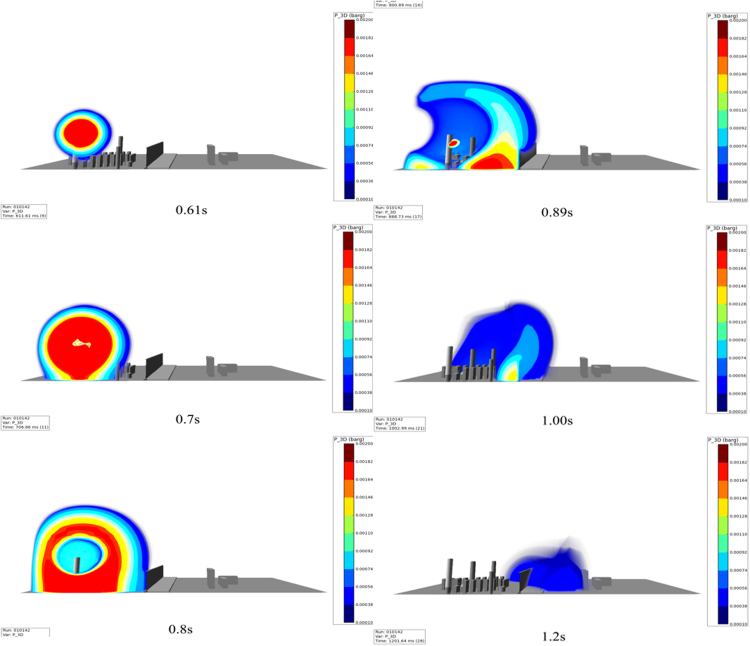
VCE with mitigation barrier.

With the developed VCE scenario, explosion simulation is conducted. Two simulation is shown in this study. One is explosion simulation without the mitigation barrier, the other is explosion simulation with the mitigation barrier. Fig. 3 shows VCE accident within 1.23 s without mitigation barrier, and Fig. 4 shows VCE accident within 1.2 s with mitigation barrier.
