# Supplementary material for: Additional data on damage reduction strategies against chemical accidents by using a mitigation barrier in Korean chemical risk management
Source: Data Brief. 2018 Aug 31;20:1753–7. doi: 10.1016/j.dib.2018.08.138 (PMC6161451; doi:10.1016/j.dib.2018.08.138)
Supplement: Supplementary file 1 — Supplementary material [file mmc1.docx]

Conflict of Interest and Authorship Conformation Form

Please check the following as appropriate:

- All authors have participated in (a) conception and design, or analysis and interpretation of the data; (b) drafting the article or revising it critically for important intellectual content; and (c) approval of the final version.
- This manuscript has not been submitted to, nor is under review at, another journal or other publishing venue.
- The authors have no affiliation with any organization with a direct or indirect financial interest in the subject matter discussed in the manuscript
- The following authors have affiliations with organizations with direct or indirect financial interest in the subject matter discussed in the manuscript:

Author’s name Affiliation

Byeonggil Lyu Department of Chemical and Biomolecular

Engineering, Yonsei University

Kwanghee Lee Department of Chemical and Biomolecular

Engineering, Yonsei University

Taejong Kim Department of Chemical and Biomolecular

Engineering, Yonsei University

Hyungtae Cho Department of Chemical and Biomolecular Engineering, Yonsei University

Seungsik Cho Department of Chemical and Biomolecular

Engineering, Yonsei University

Il Moon Department of Chemical and Biomolecular

Engineering, Yonsei University
